# The r_1_ relaxivity and T_1_ imaging properties of dendrimer-based manganese and gadolinium chelators in magnetic resonance imaging

**DOI:** 10.3389/fbioe.2022.1004414

**Published:** 2022-10-10

**Authors:** Kai Liu, Changcun Liu, Jindong Xia

**Affiliations:** ^1^ Department of Radiology, Shanghai Songjiang District Central Hospital, Shanghai, China; ^2^ Department of Nuclear Medicine, Shanghai General Hospital, Shanghai Jiao Tong University School of Medicine, Shanghai, China

**Keywords:** dendrimer, manganese, gadolinium, r_1_ relaxivity, T_1_-weighted MR imaging

## Abstract

We report the preparation and characterization of gadolinium (Gd)- or manganese (Mn)-loaded dendrimers and Gd-loaded dendrimer-entrapped gold nanoparticles (Gd-Au DENPs) to examine the relationship between the number of metal ion chelators and r_1_ relaxivity. In this study, amine-terminated fifth-generation poly(amidoamine) dendrimers (G5.NH_2_) modified with different numbers of DOTA-NHS chelators were used to chelate Gd and Mn ions. The remaining amine groups were then acetylated completely, followed by the use of materials with better r_1_ relaxivities and T_1_-weighted imaging performances as templates to synthesize Gd-Au DENPs. The Gd and Mn chelators as well as Gd-Au DENPs were characterized *via* different techniques. We show that the r_1_ relaxivity and T_1_ imaging performance increase with loading of greater numbers of Gd and Mn ions on the G5.NH_2_ and that the acetylation process affects the relaxivity and imaging properties to a certain extent. After entrapment with Au NPs, the r_1_ relaxivity and T_1_-weighted imaging performance of Gd-Au DENPs decrease with greater loading of Au NPs. This systematic study of the relaxivities and T_1_-weighted imaging performances of Gd, Mn, and Gd-Au DENP chelators are expected to be a theoretical basis for developing multifunctional dual-mode contrast agents.

## Introduction

Magnetic resonance (MR) imaging is one of the most powerful noninvasive medical imaging techniques with good spatial resolution and high sensitivity, offering superior 3D details and topographic information on soft-tissue contrast (Chan and Wong, 2007; Taboada et al., 2007; Xiao et al., 2023). To increase the signal-to-noise ratio of information acquired from normal tissues and tumors, it is necessary to use contrast agents (Aime et al., 2009). To date, various contrast agents have been used for clinical diagnosis, such as gadolinium (Gd)-based small molecular (DTPA, DOTA, NOTA) contrast agents in T_1_-weighted MR imaging and iron oxide nanoparticles (NPs)-based contrast agents in T_2_-weighted MR imaging (Miao et al., 2021; Meng et al., 2022). Unfortunately, these small-molecule-based contrast agents cannot be used for long-circulation imaging owing to their short circulation times in the blood and quick elimination from the body through urine (Prencipe et al., 2009). Although iron oxide NPs display excellent MR imaging properties and are widely used as negative MR contrast agents (Le et al., 2022; Luo et al., 2022), their clinical use has several disadvantages; in particular, negative contrast effects and magnetic susceptibility artifacts are observed, which could mislead the clinical diagnosis. Therefore, it is crucial to develop novel carriers for MR imaging contrast agents to overcome these drawbacks.

Among the macromolecular family, poly(amidoamine) (PAMAM) dendrimers are a class of highly branched, monodispersed, and synthetic macromolecules with well-defined three-dimensional architectures, composition, and abundant terminal functional groups (Shi et al., 2008). The unique structural properties of PAMAM allow their use as a platform for constructing various kinds of contrast agents, such as dendrimer-based Mn- or Gd-loaded agents for MR imaging, Gd-loaded dendrimer-entrapped gold nanoparticles (Gd-Au DENPs) for CT/MR applications (Chen et al., 2013), and ^99m^Tc labeled Mn-loaded dendrimer-based contrast agents for SPECT/MR imaging applications (Liu et al., 2012; Fan et al., 2020). The PAMAM dendrimer has been widely researched for single- and multi-mode contrast agents, but there are still some questions that have not been well resolved. First, the relationship between the r_1_ relaxivity of the chelate numbers modified onto the dendrimer and loading amount of the metal ions is not clearly understood. Second, given the same modified condition (same number of chelates and same mole amounts of metal ions), which among Mn- or Gd-based contrast agents have better r_1_ relaxivity and T_1_-weighted MR imaging performance? Third, after entrapping gold NPs, the effects of r_1_ relaxivity and T_1_-weighted MR imaging performance of Gd-loaded Au DENPs as CT/MR dual-mode contrast agents are not investigated in detail. Based on these questions, in the present study, the amine-terminated fifth-generation poly(amidoamine) (G5.NH_2_) dendrimers were first modified with 2, 2′, 2′′-(10-(2-(2, 5-dioxopyrrolidin1-aryloxy)-2-oxoethyl)-1, 4, 7,1 0-tetraazacyclododecane-1, 4, 7-triyl) triacetin acid (DOTA-NHS) in mole ratios of 1:5, 1:10, 1:20, and 1:30; then, the modified G5.NH_2_ dendrimers were used as templates to chelate Gd(III) and Mn(II). After acetylation of the remaining dendrimer terminal amines, G5.NHAc-DOTA-Gd or G5.NHAc-DOTA-Mn were formed. The formed Gd and Mn chelators were characterized thoroughly *via* different techniques, and the material with the highest r_1_ relaxivity was chosen as the template to entrap Au NPs to prepare the Gd-Au DENPs.

## Experimental methods

### Materials

Ethylenediamine core G5.NH_2_ PAMAM dendrimers (molecular weight = 26,010 g/mol) with polydispersity index values less than 1.08 were purchased from Dendritech (Midland, MI, USA). Polyethylene glycol (PEG) monomethyl ether with a carboxyl group at one end (*m*PEG-COOH) was obtained from Shanghai Yanyi Biotechnology Corporation (Shanghai, China). Gd(NO_3_)_3_.6H_2_O, MnSO_4_.H_2_O, HAuCl_4_.4H_2_O, acetic anhydride, triethylamine, and all other chemicals as well as solvents were purchased from Sinopharm Chemical Reagent Co., Ltd. (Shanghai, China). Sodium borohydride was purchased from J&K Chemical Ltd. (Shanghai, China). DOTA-NHS was purchased from CheMatech (Dijon, France). The water used in all experiments was purified using a Milli-Q Plus185 water purification system (Millipore, Bedford, MA) with a resistivity greater than 18.2 MΩ cm.

### Fabrication of G5.NH_2_ with DOTA

About 13.00 mg of G5.NH_2_ dissolved in DMSO (4 mL) was reacted with 5 molar equivalents of DOTA-NHS (1.87 mg, 2 mL in DMSO) under vigorous magnetic stirring, and purification was performed similar to that noted in our previous report (Wen et al., 2013). The reaction was stopped after 24 h to obtain the raw product G5.NH_2_-DOTA_5_. Then, G5.NH_2_-DOTA_10_, G5.NH_2_-DOTA_20_, and G5.NH_2_-DOTA_30_ were fabricated in a similar manner.

### Synthesis of G5.NH_2_-DOTA-Mn and G5.NH_2_-DOTA-Gd

**FIGURE 1:**
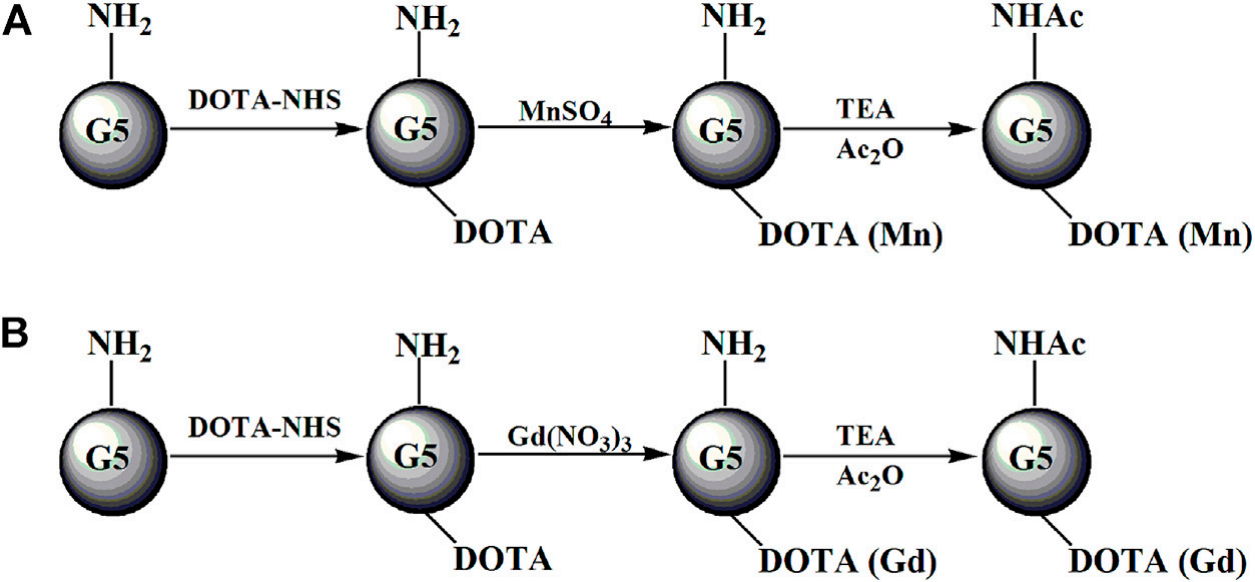
Schematic illustration of the preparation of G5.NHAc-DOTA_n_(Gd) **(A)** or G5.NHAc-DOTA_n_(Mn) **(B)**. TEA and Ac_2_O represent triethylamine and acetic anhydride, respectively.

The formed G5.NH_2_-DOTA_5_, G5.NH_2_-DOTA_10_, G5.NH_2_-DOTA_20_, and G5.NH_2_-DOTA_30_ were used as templates to chelate Mn(II) (Figure 1A) or Gd(III) (Figure 1B) ions. Aqueous MnSO_4_ (1.27 mg/mL, 1 mL in water) and Gd(NO_3_)_3_ (3.39 mg/mL, 1 mL in water) solutions with MnSO_4_/DOTA and Gd(NO_3_)_3_/DOTA in a molar ratio of 1.5:1 were added to G5.NH_2_-DOTA_5_ under vigorous stirring to chelate Mn(II) or Gd(III) ions, respectively. The reaction was performed for 24 h to obtain G5.NH_2_-DOTA_5_(Mn) or G5.NH_2_-DOTA_5_(Gd) as the products. Then, half the volumes of G5.NH_2_-DOTA_5_(Mn) and G5.NH_2_-DOTA_5_(Gd) solutions were removed for dialysis for 3 days (three times per day, 2 L of water each time) to remove the excess Mn(II) or Gd(III) ions. Six molar equivalents of the dendrimer terminal amine triethylamine (53 μL) were added to the remaining G5.NH_2_-DOTA_5_(Mn)_5_ and G5.NH_2_-DOTA_5_(Gd)_5_ solutions. After 30 min, acetic anhydride (33 μL, equal to 5 molar equivalents of the dendrimer terminal amine) was added dropwise into the solutions under vigorous magnetic stirring, and the solutions were allowed to react at room temperature with stirring for 24 h. Then, the DMSO, excess reactants, and byproducts were removed from the mixture by extensive dialysis with water (9 times, 2 L) for 3 days, followed by lyophilization to obtain the G5.NHAc-DOTA_5_(Mn) and G5.NHAc-DOTA_5_(Gd). The G5.NH_2_-DOTA_10_(Mn), G5.NH_2_-DOTA_20_(Mn), G5.NH_2_-DOTA_30_(Mn), G5.NH_2_-DOTA_10_(Gd), G5.NH_2_-DOTA_20_(Gd), G5.NH_2_-DOTA_30_(Gd), and their acetylated materials were also formed similarly.

### Synthesis of (Au^0^)_n_-G5.NHAc-*m*PEG_15_-DOTA_30_(Gd)

**FIGURE 2:**
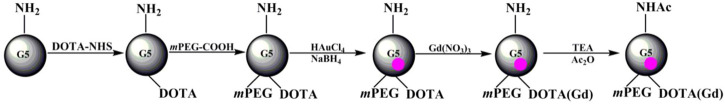
Schematic illustration of the preparation of (Au^0^)_n_-G5.NHAc-*m*PEG-DOTA_30_(Gd). TEA and Ac_2_O represent triethylamine and acetic anhydride, respectively.

About 13.00 mg of G5.NH_2_ dissolved in DMSO (4 mL) was reacted with 30 molar equivalents of DOTA-NHS (11.22 mg, 5 mL in DMSO) under vigorous magnetic stirring (Figure 2). The reaction was stopped after 24 h to obtain G5.NH_2_-DOTA_30_ as the raw product. Then, 15.00 mg of *m*PEG-COOH dissolved in 5 mL DMSO and 15 molar equivalents of G5.NH_2_ was reacted with EDC (14.38 mg in 2 mL DMSO) for 15 min at room temperature. Next, NHS (8.63 mg, in 1 mL DMSO) was added to the above solution under vigorous magnetic stirring for 3 h. The EDC/NHS-activated *m*PEG-COOH was then added dropwise into the DMSO solution of the raw product of G5.NH_2_-DOTA_30_ under vigorous magnetic stirring. The reaction was continued for 3 days to obtain G5.NH_2_-DOTA_30_-*m*PEG_15_ conjugates as the raw products. Then, HAuCl_4_ solution (5 mg/mL, 2.06 mL in water) was added under vigorous stirring. After 1 h, an icy cold NaBH_4_ solution (4.73 mg/mL, 1 mL in water/methanol, v/v = 2:1) with 5 molar equivalents of the gold salt was added to the gold salt/dendrimer mixture under vigorous stirring. The solution turned a deep-red color after the addition of NaBH_4_, and the stirring was continued for 3 h to complete the reaction. Then, acetylation of the excess terminal amines was performed similar to the process in our previous work (Liu et al., 2012). The mixture was purified by dialysis as described previously to remove the excess DMSO and other reagents. The Au DENPs were then purified and dried by lyophilization.

### Characterization techniques

^1^H NMR spectra were recorded with a Bruker DRX 400 nuclear magnetic resonance spectrometer. Samples were dissolved in D_2_O before the measurements. The size and morphology of the Gd-Au DENPs were characterized by using a JEOL 2010F analytical electron microscope (JEOL, Japan) operating at 200 kV. Transmission electron microscopy (TEM) samples were prepared by the deposition of a dilute particle suspension (1 mg/mL, 5 μL) onto a carbon-coated copper grid and air dried before measurements. For each sample, at least 100 NPs from different TEM images were randomly selected and measured using ImageJ software (http://www.rsb.info.nih.gov/ij/download.html) to assess the average size and size distribution. The compositions of Mn, Gd, and Au of the formed materials were determined by inductively coupled plasma optical emission spectroscopy (ICP-OES, Leeman Prodigy, USA). The surface potentials of each material before and after acetylation were measured using a Zetasizer Nano ZS system (Worcestershire, UK) equipped with a standard 633 nm laser. The r_1_ relaxivity and T_1_-weighted images of the Gd and Mn chelates were obtained using a 0.5 T NMI20 equipment (Newman, China) with a wrist receiver coil.

## Results and discussion

### Synthesis and characterization of G5.NH_2_-DOTA

The ^1^H NMR technique was used to investigate the actual number of DOTA units conjugated onto each G5.NH_2_ molecule, as in our previous work (Supplementary Figure S1;Wen et al., 2013). After conjugation of different ratios of DOTA-NHS, the numbers of remaining dendrimer terminal amine groups in each of the G5.NH_2_ molecules were estimated to be 105.8, 102.5, 99.6, and 90.1, and the numbers of DOTA units per G5.NH_2_ molecule were calculated as 4.2, 7.5, 10.4, and 19.9 (Supplementary Table S1); these numbers are slightly lower than the theoretical values of 5, 10, 20, and 30 based on the initial molar feed ratios.

### Fabrication and characterization of G5.NHAc–DOTA(Mn) and G5.NHAc-DOTA(Gd)

The numbers of Gd(III) and Mn(II) ions complexed with each dendrimer were measured as shown in Supplementary Table S2. It is clear that the number of ions per G5.NH_2_ molecule of the Gd-based chelate is slightly higher than the DOTA number on the surface of the dendrimer, which is consistent with the observations in our previous work (Wen et al., 2013). The reason for this is likely the fact that besides the Gd(III) ions chelated within the DOTA ligands on the surfaces of the G5 dendrimers, small portions of the Gd(III) ions are complexed with the interior tertiary amine groups of the dendrimer. In contrast, the number of Mn(II) ions complexed with each G5.NH_2_ molecule was estimated to be 23.3 when the initial molar feed ratio of the DOTA/dendrimer was 30:1; this is less than the theoretical value of 30 attached DOTA moieties on each dendrimer. Compared with the Mn-based chelates, the Gd-based chelates thus have better chelating abilities.

To utilize the G5.NH_2_ modifications to Gd- and Mn-based chelates in biological applications, it is necessary to explore their cytotoxicities before and after acetylation. Our previous report shows that the G5.NH_2_ displays significant cytotoxicity because of more than 100 amino groups on the surface of the G5.NH_2_ dendrimer. To investigate the changes in the zeta potentials of each of the materials before and after acetylation, the zeta potential of each material was measured. As shown in Supplementary Table S3, the zeta potential of each material decreased sharply after acetylation. This may be attributed to the redundant amino groups of G5.NH_2_ being changed into acetamide groups with acetic anhydride through acetylation.

### Synthesis and characterization of {(Au^0^)_n_-G5.NHAc-*m*PEG_15_-DOTA_3_(Gd)} DENPs

**FIGURE 3:**
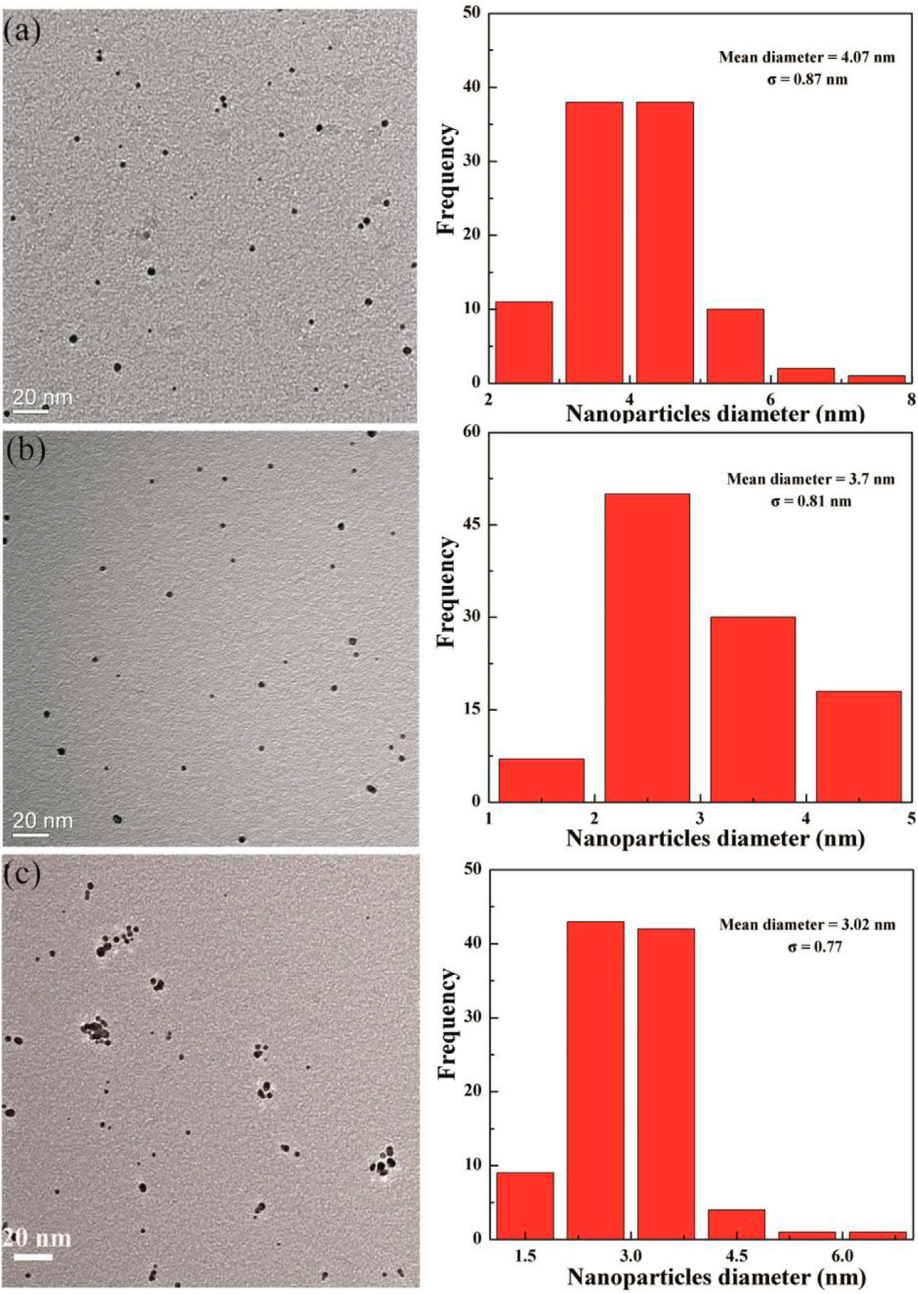
Transmission electron microscopy images of (Au^0^)_50_G5.NHAc-mPEG-DOTA_30_(Gd) **(A)**, (Au^0^)_75_ G5.NHAc-*m*PEG-DOTA_30_(Gd) **(B)**, and (Au^0^)_100_G5.NHAc-*m*PEG-DOTA_30_(Gd) **(C)** DENPs.

The size and morphology of Au DENPs of different compositions were characterized by TEM (Figure 3). The diameters of the **(**Au^0^)_50_-G5.NHAc-*m*PEG_15_-DOTA_30_-Gd, **(**Au^0^)_75_-G5.NHAc-*m*PEG_15_-DOTA_30_-Gd, and (Au^0^)_100_-G5.NHAc-*m*PEG_15_-DOTA_30_-Gd DENPs were 4.1, 3.7, and 3.0 nm, respectively. The diameter of the Au DENPs increased slightly with the high dendrimer/Au salt molar ratio, which is very close to that noted in our previous study (Wen et al., 2013).

### T_1_-weighted imaging and r_1_ relaxivity of Mn-based and Gd-based chelates

**FIGURE 4:**
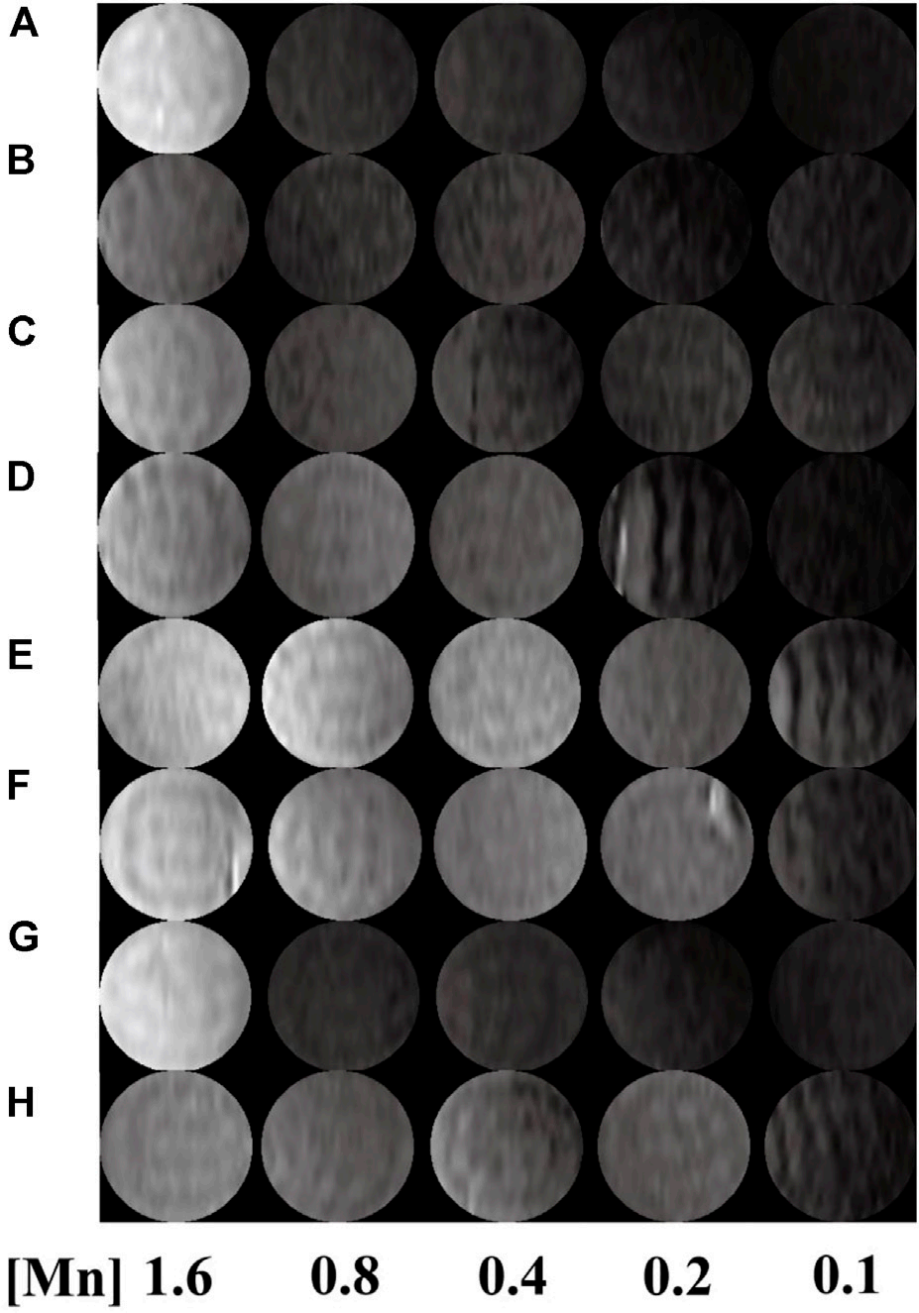
T_1_-weighted images of G5.NH_2_-DOTA_5_(Mn), G5.NH_2_-DOTA_10_(Mn), G5.NH_2_-DOTA_20_(Mn), and G5.NH_2_-DOTA_30_(Mn) **(A, C, E, G)** before and **(B, D, F, H)** after acetylation as functions of Mn^2+^ concentrations.

**FIGURE 5:**
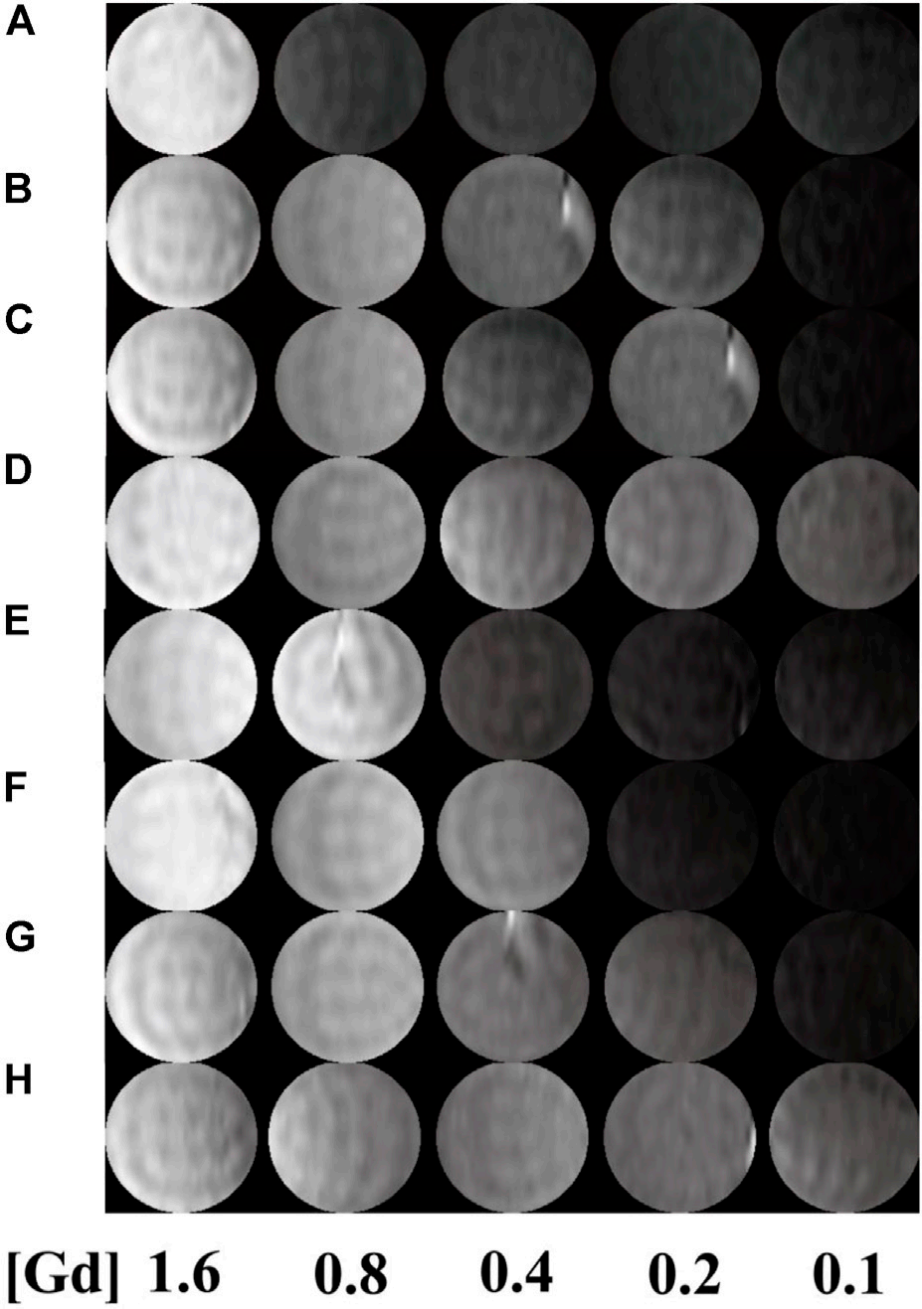
T_1_-weighted images G5.NH_2_-DOTA_5_(Gd), G5.NH_2_-DOTA_10_(Gd), G5.NH_2_-DOTA_20_(Gd), and G5.NH_2_-DOTA_30_(Gd) **(A, C, E, G)** before and **(B, D, F, H)** after acetylation as functions of Gd^3+^ concentrations.

T_1_-weighted imaging was conducted to verify the potential of the formed Mn-based (Figure 4) as well as Gd-based (Figure 5) materials as MR contrast agents. The proton longitudinal relaxation times (T_1_) of these sixteen samples in water were measured with a 0.5 T NMI20 MRI system (Newman, China) with Mn^2+^ and Gd^3+^ concentrations of 0.1, 0.2, 0.4, 0.8, and 1.6 mM. As shown in Figure 3 and Figure 4, signal enhancements are observed in the T_1_-weighted images in a number-dependent manner with increases in the numbers of DOTA-Mn and DOTA-Gd ions per G5.NH_2_. For the Mn-chelates, the signal enhancements in the T_1_-weighted images were inconspicuous even though the number of DOTA-Mn increased to 20. We found that the signals of the Mn chelates weakened after acetylation. On the contrary, the signals of the Gd chelates increased slightly after acetylation. As shown in Table 1, the r_1_ relaxivity of the DOTA-Mn increased from 1.16 to 2.54 mM^−1^s^−1^ with increase in number from 5 to 30 per G5.NH_2_. After acetylation, the r_1_ relaxivity of the DOTA-Mn decreased obviously from 1.90 to 0.55 mM^−1^s^−1^. The r_1_ relaxivity results follow the same trends as the T_1_-weighted images. The r_1_ relaxivity of the Gd-based chelates increased from 5.77 to 7.69 mM^−1^s^−1^ with increase in the numbers of DOTA-Gd from 5 to 30 per G5.NH_2_. However, the r_1_ relaxivities of the Gd-based chelates increased from 7.06 to 9.77 after acetylation, which is different from those of the Mn-based chelates. We speculate that the increased r_1_ relaxivities of the Gd- and Mn-based chelates may be due to the additional DOTA conjugates on the G5.NH_2_ to form nanoclusters as it has been noted in literature that the cluster structure could promote water exchange rate to increase the r_1_ relaxivity (Matsumoto and Jasanoff, 2008).

**TABLE 1:** Linear fitting of the r_1_ relaxivities of G5.NH_2_-DOTA(Mn) and G5.NH_2_-DOTA(Gd) before and after acetylation as functions of Mn and Gd concentrations, respectively.

Sample	Before acetylation (mM^−1^s^−1^)	After acetylation (mM^−1^s^−1^)
G5.NH_2_-DOTA_5_(Mn)	1.16	0.55
G5.NH_2_-DOTA_10_(Mn)	2.66	1.90
G5.NH_2_-DOTA_20_(Mn)	2.52	1.66
G5.NH_2_-DOTA_30_(Mn)	2.54	1.26
G5.NH_2_-DOTA_5_(Gd)	5.77	7.06
G5.NH_2_-DOTA_10_(Gd)	6.92	9.24
G5.NH_2_-DOTA_20_(Gd)	7.59	9.67
G5.NH_2_-DOTA_30_(Gd)	7.69	9.77

### T_1_-weighted imaging and r_1_ relaxivity of Gd-Au DENPs

**FIGURE 6:**
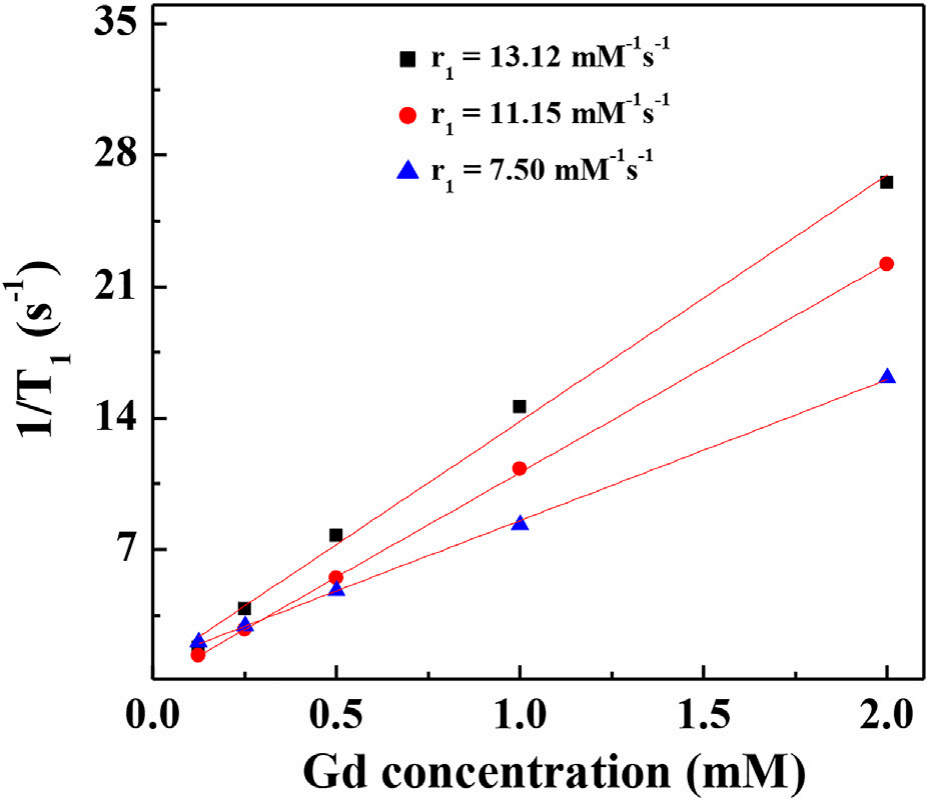
Linear fitting 1/T_1_ of (Au^0^)_50_G5.NHAc-*m*PEG-DOTA_30_(Gd) (black square), (Au^0^)_75_ G5.NHAc-*m*PEG-DOTA_30_(Gd) (red circle), and (Au^0^)_100_G5.NHAc-*m*PEG-DOTA_30_(Gd) (blue triangle) DENPs at Gd concentrations of 2, 1, 0.5, 0.25 and 0.125 mM.

**FIGURE 7:**
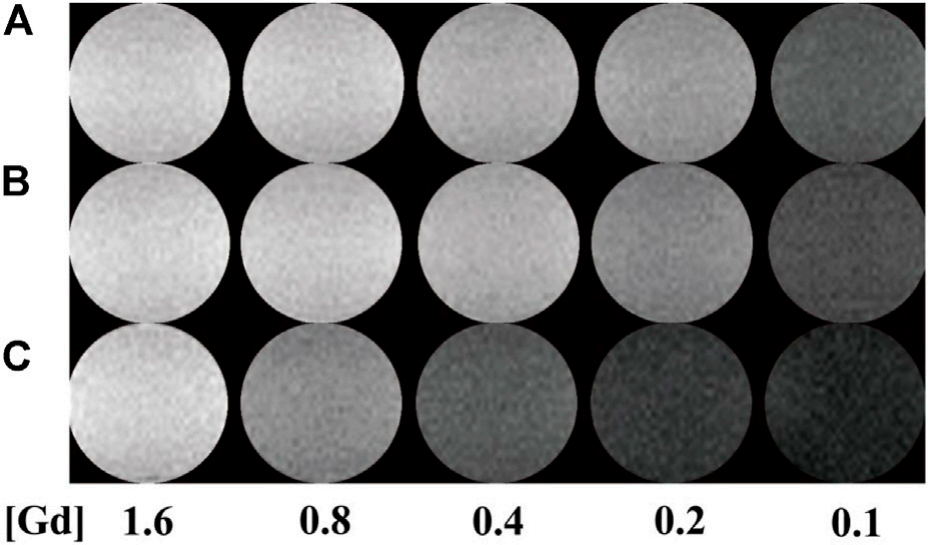
T_1_-weighted images of **(A–C)** (Au^0^)_50_G5.NHAc-*m*PEG-DOTA_30_(Gd), (Au^0^)_75_ G5.NHAc-*m*PEG-DOTA_30_(Gd), and (Au^0^)_100_G5.NHAc-*m*PEG-DOTA_30_(Gd) DENPs at Gd concentrations of 1.6, 0.8, 0.4, 0.2, and 0.1 mM.

It has been reported that Au NPs could be used as CT contrast agents because they have good biocompatibility. To investigate the relationship between the number of gold NPs and r_1_ relaxivity of the Gd-based chelates, we fabricated different G5:Au mole ratios of 1:50, 1:75, and 1:100 NPs and measured the r_1_ relaxivities (Figure 6) of Gd-Au DENPs as well as obtained their T_1_-weighted images (Figure 7). Figure 4 shows that with the increase in the amount of gold atoms per G5.NH_2_, the r_1_ relaxivity decreased from 13.11 to 7.50 mM^−1^s^−1^. This is attributed to the fact that the gold NPs being entrapped in the interior of the G5.NH_2_ primary cavity structure changed and that the G5.NH_2_ molecule became tight, leading to reduced water exchange rate between its interior and exterior.

## Conclusion

We systematically investigated the relationship between r_1_ relaxivity and number of metal ions per dendrimer in this study. T_1_ relaxometry measurements show that the formed G5.NHAc-DOTA(Gd) NPs have an r_1_ relaxivity of 9.77 mM^−1^ s^−1^ when the number of DOTA is 30. Compared with dendrimer-based Gd chelators, the Mn-based materials show lower r_1_ relaxivity and poor T_1_ imaging properties for the same number of DOTA units. When the mole ratio of gold to G5 is as high as 100:1, the r_1_ relaxivity of G5.NHAc-DOTA_30_-Gd decreased to 7.50 mM^−1^s^−1^ and T_1_ imaging property was weakened. With appropriate tuning of the number of ions per G5 and the Gd/Au composition, the formed Gd-based or Gd/Au NPs may be applied in dual-mode MR/CT imaging and diagnosis of particular diseases (e.g., cancer) with high accuracies.

## Data Availability

The original contributions presented in the study are included in the article/Supplementary Material, and further inquiries can be directed to the corresponding author.
