# Supplementary material for: The r1 relaxivity and T1 imaging properties of dendrimer-based manganese and gadolinium chelators in magnetic resonance imaging
Source: Front Bioeng Biotechnol. 2022 Oct 10;10:1004414. doi: 10.3389/fbioe.2022.1004414 (PMC9589045; doi:10.3389/fbioe.2022.1004414)
Supplement: Supplementary file 1 [file DataSheet1.doc]

**Supporting Information**

**The r_1_** **relaxivity and T_1_ imaging property of dendrimer-based manganese and gadolinium chelators applied for MR imaging**

Kai Liu^1^, Changcun Liu^2^, Jindong Xia*^1^

^1^ Department of Radiology, Shanghai Songjiang District Central Hospital, Shanghai 201600, P. R. China

^2^ Department of Nuclear Medicine, Shanghai General Hospital, Shanghai Jiao Tong University School of Medicine, Shanghai 201620, P. R. China

Table S1. The theoretical and actual number of DOTA per G5.NH_2_.

| G5: DOTA (mol/mol) | Theoretical value | Actual value |
| --- | --- | --- |
| 1:5 | 5 | 4.2 |
| 1:10 | 10 | 7.5 |
| 1:20 | 20 | 10.4 |
| 1:30 | 30 | 19.9 |

Table S2. The number of Gd(III) and Mn(II) per G5.NH_2_.

| G5 : DOTA (mol/mol) | The value of Gd(III) | The value of Mn(II) |
| --- | --- | --- |
| 1:5 | 4.2 | 3.8 |
| 1:10 | 11.6 | 12.7 |
| 1:20 | 20.9 | 17.9 |
| 1:30 | 31.9 | 23.3 |

Table S3. Zeta-Potential values of the formed G5.NH_2_-DOTA(Mn) and G5.NH_2_-DOTA(Gd) with different metal compositions. Data were provided with mean ± standard deviation (n = 3).

| Sample | Before acetylation (mV) | After acetylation (mV) |
| --- | --- | --- |
| G5.NH_2_-DOTA_5_(Mn) | 43.3 ± 3.1 | 21.3 ± 0.3 |
| G5.NH_2_-DOTA_10_(Mn) | 41.3 ± 3.7 | 11.6 ± 1.9 |
| G5.NH_2_-DOTA_20_(Mn) | 34.7 ± 3.0 | 12.6 ± 2.4 |
| G5.NH_2_-DOTA_30_(Mn) | 35.3 ± 2.7 | 12.7 ± 1.4 |
| G5.NH_2_-DOTA_5_(Gd) | 41.1 ± 6.1 | 18.7 ± 2.2 |
| G5.NH_2_-DOTA_10_(Gd) | 46.2 ± 4.3 | 13.4 ± 4.9 |
| G5.NH_2_-DOTA_20_(Gd) | 35.1 ± 0.7 | 16.6 ± 1.3 |
| G5.NH_2_-DOTA_30_(Gd) | 36.9 ± 3.6 | 18.4 ± 2.3 |

Table S4. The Zeta-Potential of the formed (Au^0^)_50_G5.NHAc-*m*PEG-DOTA_30_(Gd), (Au^0^)_75_ G5.NHAc-*m*PEG-DOTA_30_(Gd) and (Au^0^)_100_G5.NHAc-*m*PEG-DOTA_30_(Gd) DENPs. Data were provided with mean ± standard deviation (n = 3).

| Sample | Before acetylation (mV) | After acetylation (mV) |
| --- | --- | --- |
| (Au^0^)_50_G5.NHAc-*m*PEG-DOTA_30_(Gd) | 38.6 ± 1.9 | 15.6 ± 0.6 |
| (Au^0^)_75_ 5.NHAc-*m*PEG-DOTA_30_(Gd) | 33.5 ± 2.4 | 12.4 ± 0.4 |
| (Au^0^)_100_G5.NHAc-*m*PEG-DOTA_30_(Gd) | 31.1 ± 1.8 | 10.9 ± 1.8 |

Table S5. The hydrodynamic size of the formed (Au^0^)_50_G5.NHAc-*m*PEG-DOTA_30_(Gd), (Au^0^)_75_ G5.NHAc-*m*PEG-DOTA_30_(Gd) and (Au^0^)_100_G5.NHAc-*m*PEG-DOTA_30_(Gd) DENPs. Data were provided with mean ± standard deviation (n = 3).

| Sample | Before acetylation (nm) | After acetylation (nm) |
| --- | --- | --- |
| (Au^0^)_50_G5.NHAc-*m*PEG-DOTA_30_(Gd) | 62.8 ± 7.9 | 64.4 ± 1.7 |
| (Au^0^)_75_ 5.NHAc-*m*PEG-DOTA_30_(Gd) | 69.7 ± 4.3 | 71.5 ± 3.1 |
| (Au^0^)_100_G5.NHAc-*m*PEG-DOTA_30_(Gd) | 70.4 ± 5.8 | 72.1 ± 1.6 |


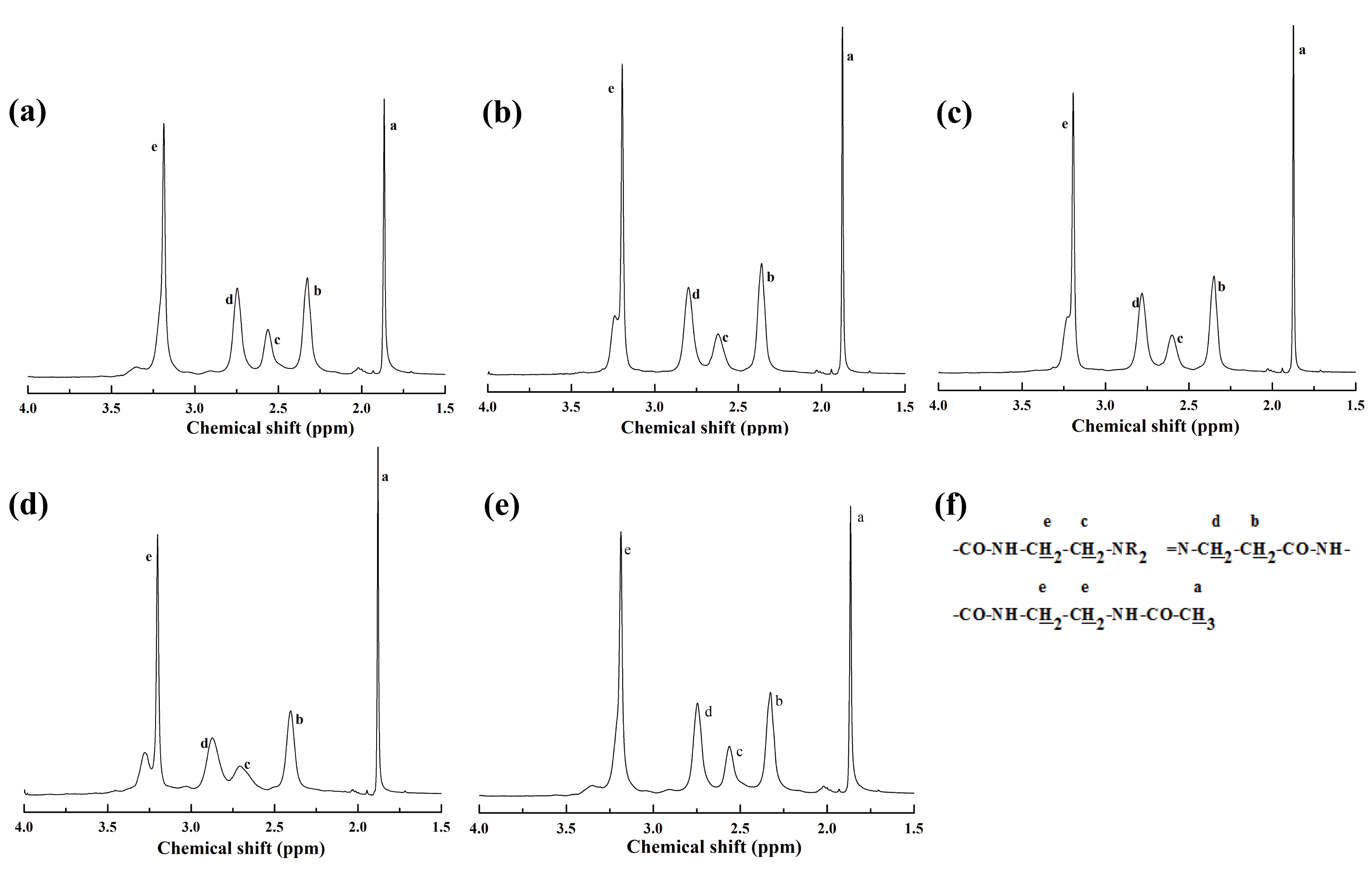


**Figure S1.** ^1^H NMR spectra of acetylated G5.NH_2_ (a), G5.NH_2_-DOTA_5_ (b), G5.NH_2_-DOTA_10_ (c), G5.NH_2_-DOTA_20_ (d), G5.NH_2_-DOTA_30_ (e) and the dendrimer structure used for NMR peak assignment (f)
